# Immunomodulatory Effect of *Stichopus japonicus* Acid Mucopolysaccharide on Experimental Hepatocellular Carcinoma in Rats

**DOI:** 10.3390/molecules18067179

**Published:** 2013-06-19

**Authors:** Yang Song, Shou-Jie Jin, Lian-Hua Cui, Xiao-Jun Ji, Fu-Guo Yang

**Affiliations:** 1 Institute of Nutrition, Qingdao University Medical College, Qingdao 266021, Shandong, China; E-Mails: qdjinshj@163.com (S.-J.J.); qdlhcui@163.com (L.-H.C.); yfuguo@126.com (F.-G.Y.); 2 The Affiliated Hospital of Qingdao University Medical College, Qingdao 266021, Shandong, China; E-Mail: jixiaojun123@126.com

**Keywords:** hepatocellular carcinoma, *Stichopus japonicus* acid mucopolysaccharide, macrophage, natural killer cell, T lymphocyte subsets, proliferating cell nuclear antigen, p21 expression

## Abstract

*Stichopus japonicus* acid mucopolysaccharide (SJAMP) is an important biologically active compound that can be extracted from the body wall of the sea cucumber. The present study investigated the anti-tumor and immunomodulatory effects of SJAMP in an experimental hepatocellular carcinoma (HCC) model in rats. Three doses of SJAMP (17.5 mg/kg, 35 mg/kg, and 70 mg/kg administered 5 days/week via oral gavage) were given to rats with diethylnitrosamine (DEN)-induced HCC. SJAMP treatment significantly inhibited DEN-induced HCC by reducing both the number and mean volume of nodules, decreasing serum α-fetoprotein (AFP) levels and proliferating cell nuclear antigen (PCNA) expression in liver, and increasing p21 expression. Furthermore, SJAMP decreased the serum levels of ALT, AST, GGT and TNF-α and increased serum IL-2. SJAMP administration also improved indices of spleen and thymus function and improved both macrophage phagocytosis and NK cell-mediated tumoricidal activity. Moreover, CD3^+^ and CD4^+^ T lymphocyte levels recovered significantly and the CD4^+^/CD8^+^ T cell ratio normalized in a dose-dependent manner. In conclusion, SJAMP effectively inhibited the growth of HCC through the stimulation of immune organs and tissue proliferation, leading to the enhancement of cellular immunity pathways in rats.

## 1. Introduction

Hepatocellular carcinoma (HCC) is one of the most lethal human cancers and the third leading cause of cancer mortality worldwide, resulting in some 600,000 deaths annually [1]. Despite significant advances in surgery and radiotherapy, not all patients respond fully to therapeutic intervention. Furthermore, traditional chemotherapeutic drugs have several deficiencies including poor specificity, side effects and low tolerability. The enhancement of the host’s immune response has been recognized as a possible means of inhibiting tumor growth without harm to the host [2]. Therefore, the study of novel natural biochemical materials that have anti-tumor activity and immunostimulatory properties has piqued the interests of many researchers. 

Sea cucumbers are marine animals that are important as a human food source, particularly in some parts of Asia. They are typically soft-bodied echinoderms comprising a diverse group of flexible, elongated, worm-like organisms with leathery skin and a gelatinous body that resembles a cucumber [3]. A multitude of harvestable sea cucumber species have been developed to meet the growing global demand for their food and pharmaceutical properties. *Stichopus japonicus* acid mucopolysaccharide (SJAMP) is an important biologically active compound that is extracted from the body wall of the sea cucumber *Stichopus japonicus*. SJAMP is made up of galactosamine, hexuronic acid, fucose and sulfuric acid with a proportion of 1:1:1:4 [4,5,6]. SJAMP has multiple pharmacologic properties, including anti-tumor, immunologic regulation, anticoagulant and antiviral properties [3]. Previous studies have shown that SJAMP has protective effects against several cancers and inhibits the proliferation of malignant cells through the induction of apoptosis [7,8]. To date, studies of the anti-tumor effect of SJAMP *in vitro* have mainly focused on several different types of tumor cells, including HeLa, lung cancer, stomach cancer and liver cancer cells [4,7,8]. However, the mechanisms of the anti-tumor activity of SJAMP remain largely unexplored.

There is a close relationship between immune functional status and the occurrence and progression of tumors [9,10,11,12]. Tumor cells that develop mechanisms against normal immune response, they may become pathogenic tumors, while down-regulation of the immune response in tumor-bearing patients may result in further tumor development, up-regulaton has been shown to be a useful therapeutic approach [13]. Immunotherapy is a relatively novel approach to tumor therapy. However, numerous immune tolerogenic factors induced by viral infection or a chronic inflammatory response during hepatocyte carcinogenesis may accumulate, facilitating an aggressive and effective counterattack against the anti-HCC immunity of the host [14]. In developing tumors anti-tumorigenic and pro-tumorigenic immune and inflammatory mechanisms coexist [15]. Although the proinflammatory signaling cascades that mediate the pro-tumorigenic effects of inflammation are often activated during HCC development [15,16], immunomodulatory agents including those to release immune suppression and boost anti-HCC immunity are promising [14].

In our current study, we therefore investigated the anti-tumor and immunomodulatory activities of SJAMP in a diethylnitrosamine (DEN)-induced HCC rat model. The results may help provide an experimental and theoretical basis for the study of the anti-tumor effects of SJAMP.

## 2. Results and Discussion

### 2.1. Growth Conditions of the Rats in the Feeding Process

Rats in the normal control group produced normal urine and showed significant gains in weight, good gloss and high-quality fur. After eight weeks of feeding to produce long-term exposure to DEN, rats in the tumor control group displayed signs of poor health, including apathy, dull coats, gloss loss, loss of appetite, reduced activity and slow growth. Rats in the SJAMP intervention groups were distinctly better than those in the tumor control group. During the course of the experiment, three rats died in the tumor control group, and one rat died in each of the low-SJAMP-dose group and the medium-SJAMP-dose groups. Table 1 shows the body weight changes of the animals in each group. There were no significant differences in mean body weight among the groups at baseline (*p* > 0.05). At the termination of the experiment (at the 16th week), the final mean body weight of the rats in the DEN-induced tumor control group was significantly lower than the normal control group (*p* < 0.05). The final mean body weight of the rats in the high-SJAMP-dose group was significantly higher than in the tumor control group (*p* < 0.05). The low- or medium-SJAMP-dose groups also had increased mean final body weights compared to the tumor control group, although the increase did not reach statistical significance.

**Table 1:** Weight change of rats over the experimental period (x ± SD).

Groups	Number (n)	Deaths (n)	Pre-experimental (g)	Post-experimental (g)
Normal control	10	0	197.4 ± 20.3	566.9 ± 73.1
Tumor control	10	3	202.4 ± 27.2	441.3 ± 50.3 ^a^
Low SJAMP dose (17.5 mg/kg)	10	1	197.0 ± 22.5	460.7 ± 52.4 ^a^
Medium SJAMP dose (35 mg/kg)	10	1	192.7 ± 18.9	476.6 ± 41.8 ^a^
High SJAMP dose (70 mg/kg)	10	0	208.5 ± 16.9	509.5 ± 26.7 ^ab^

^a^ *p* < 0.05 *vs.* normal control group; ^b^ *p* < 0.05 *vs.* tumor control group.

### 2.2. The Inhibitory Effect of SJAMP on Tumor Growth in Rats with DEN-Induced HCC

**Figure 1:**
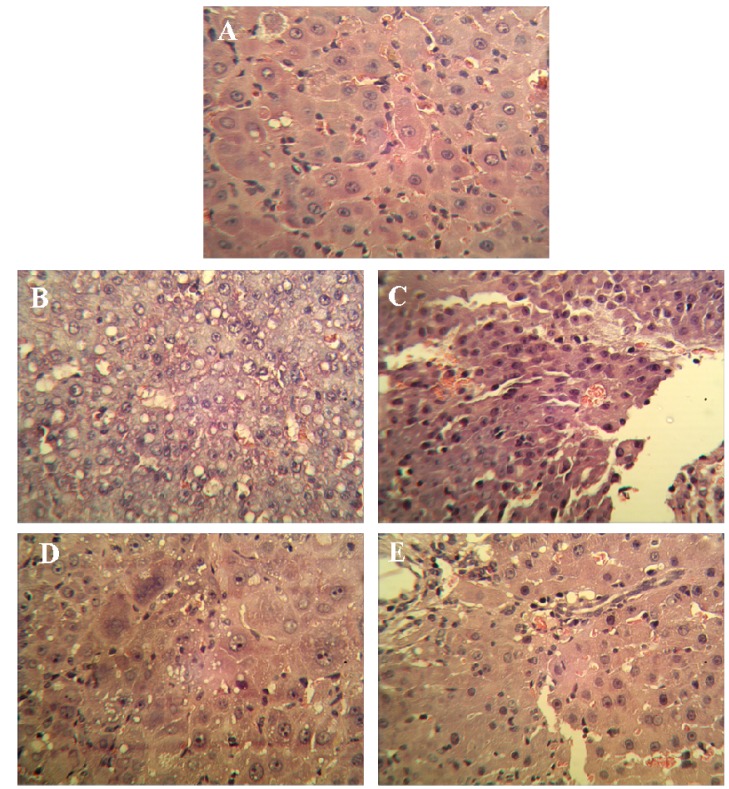
H&E-stained liver tissue biopsies from rats by experimental group (magnification factor × 400). (**A**) normal control group; (**B**) tumor control group; (**C**) low-SJAMP-dose group (17.5 mg/kg); (**D**) medium-SJAMP-dose group (35 mg/kg); (**E**) high-SJAMP-dose group (70 mg/kg).

Visible nodules were found in all tumor control and SJAMP intervention groups. Compared to the tumor control group, medium (35 mg/kg) and high (70 mg/kg) SJAMP dose significantly reduced the number of nodules greater than 3 mm and 5 mm in diameter, respectively (Table 2; *p* < 0.05). The low SJAMP dose also decreased the number of nodules compared with the tumor control group, but the reduction did not reach statistical significance. The mean volume of the largest nodules in the SJAMP intervention groups were significantly less than in the tumor control group (*p* < 0.05). Figure 1 shows the histopathological changes in the liver tissues of the rats in the different groups. In the livers from normal control group rats, hepatic lobules were structurally intact, hepatic cords were arranged in neat rows, and nuclei were clearly visible (Figure 1A). Livers from tumor control group rats showed characteristics of Hepatocellular carcinoma or neoplasm, including obvious nodular liver lesions, irregularly shaped or spindle-shaped cell changes, plentiful tumor vessels, varying degrees of fatty degeneration, hyperchromatic and prominent nuclei and mitosis (Figure 1B). There were different degrees of pathological changes in the liver tissues of the rats in the SJAMP intervention groups. No significant differences between the low-SJAMP-dose group (Figure 1C) and the tumor control group were observed (Figure 1B). However, the hepatic lobules were arranged neatly with only slight steatosis and floating cancer cells in the medium-SJAMP-dose (Figure 1D) and high-SJAMP-dose groups (Figure 1E). 

**Table 2:** Effect of SJAMP intervention on liver tumor incidence (x ± SD).

Groups	Number (n)	Average number of nodules/nodule-bearing liver (>3 mm)	Average number of nodules/nodule-bearing liver (>5 mm)	Maximum nodule volume (mm^3^)
Normal control	10	-	-	-
Tumor control	7	38.1 ± 13.5	4.3 ± 1.1	178.77 ± 58.56
Low SJAMP dose (17.5 mg/kg)	9	37.8 ± 12.3	3.9 ± 0.8	116.14 ± 28.18 ^a^
Medium SJAMP dose (35 mg/kg)	9	30.4 ± 8.1 ^a^	3.1 ± 0.6 ^a^	89.65 ± 25.42 ^a^
High SJAMP dose (70 mg/kg)	10	26.1 ± 11.1 ^ab^	2.6 ± 1.1 ^ab^	66.37 ± 18.00 ^ab^

^a^ *p* < 0.05 *vs.* tumor control group; ^b^ *p* < 0.05 *vs.* low-SJAMP-dose group.

DEN-induced HCC in animals is one of the best-characterized experimental models of HCC, and it is widely used to investigate hepatocarcinogenesis and to screen potential anti-HCC compounds [17,18,19]. Our results clearly indicate that SJAMP was able to reduce not only the number of DEN-induced HCC lesions but also the volume of the largest nodules in each individual rat with HCC. This finding is of critical importance because a liver affected with HCC usually contains multiple malignant foci, and the existence of multiple small foci is the most common factor leading to the recurrence of HCC. Higher doses of SJAMP also led to greater anti-tumor effects, further supporting the effectiveness of SJAMP as an anti-HCC agent.

### 2.3. The Effect of SJAMP on Liver Injury and Cancer Markers in Rats with DEN-Induced HCC

**Figure 2:**
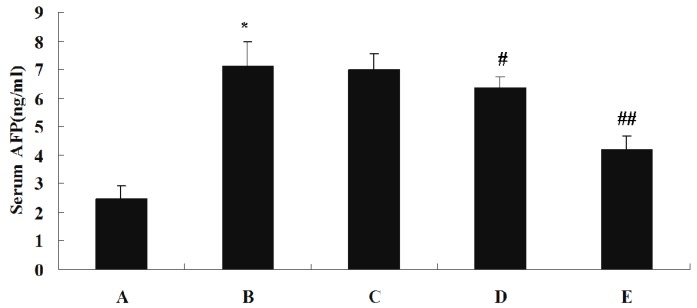
Effect of SJAMP on the levels of serum AFP in rats with DEN-induced HCC. (A) normal control group; (B) tumor control group; (C) low-SJAMP-dose group (17.5 mg/kg); (D) medium-SJAMP-dose group (35 mg/kg); (E) high-SJAMP-dose group (70 mg/kg). Data are expressed as the means ± SD. * *p* < 0.01 *vs.* normal control group; ^#^ *p* < 0.05, ^##^ *p* < 0.01 *vs.* tumor control group, respectively.

The effect of SJAMP on liver injury is shown in Table 3. Levels of serum alanine aminotransferase (ALT), aspartate aminotransferase (AST) and γ-glutamyltranspeptidase (GGT) were significantly increased in the DEN-induced tumor control group compared to the normal control group (*p* < 0.01). These factors were significantly decreased in the SJAMP intervention groups relative to the normal control group (*p* < 0.01), and showed a clear dose-response relationship. Figure 2 depicts the levels of the tumor marker Alpha-fetoprotein (AFP) in serum as assessed by an ELISA assay. While in the DEN-induced tumor control group there was a marked increase in AFP level compared to the normal control group, the levels of AFP were significantly reduced in the medium-SJAMP-dose group and high-SJAMP-dose group compared with the tumor control group (*p* < 0.05 and *p* < 0.01, respectively). The low-SJAMP-dose group also reduced the serum AFP level, but this was not statistically significant relative to the tumor control group.

The increase in serum ALT, AST and GGT indicated that DEN may induce acute liver injury. During carcinogenesis, these enzymes could be used as biomarkers of HCC response to therapy. As seen in the present study, treatment with SJAMP significantly reduced serum ALT, AST and GGT levels compared to DEN-treated animals, which suggests that SJAMP may be a potential protective agent against the liver toxicity of DEN.

AFP has been widely used as a clinical marker in the diagnosis and monitoring of HCC. In our study, there was a marked increase in serum AFP level in DEN-induced tumor control group animals. This finding is consistent with previous report that DEN-induced HCC in rats led to an increase in serum AFP [20]. The medium and high doses of SJAMP significantly reduced serum AFP level, suggesting that SJAMP might deaden lesion of the liver and delay the DEN-induced HCC in rats.

**Table 3:** Effect of SJAMP on the levels of serum ALT, AST and GGT in rats with DEN-induced HCC (x ± SD).

Groups	Number (n)	ALT (U/L)	AST (U/L)	GGT (U/L)
Normal control	10	63.44 ± 10.42	139.00 ± 27.92	7.70 ± 5.76
Tumor control	7	96.57 ± 28.62 ^a^	394.86 ± 34.01 ^a^	126.71 ± 18.37 ^a^
Low SJAMP dose (17.5 mg/kg)	9	202.67 ± 25.59 ^b^	301.11± 30.84 ^b^	98.67 ± 16.40 ^b^
Medium SJAMP dose (35 mg/kg)	9	223.00 ± 29.80 ^b^	260.11 ± 27.72 ^b^	96.67 ± 11.34 ^b^
High SJAMP dose (70 mg/kg)	10	166.70 ± 23.25 ^b^	239.80 ± 34.58 ^b^	86.80 ± 16.38 ^b^

^a^ *p* < 0.01 *vs.* normal control group; ^b^ *p* < 0.01 *vs.* tumor control group.

### 2.4. The Effect of SJAMP on the Expression of PCNA and p21 in Rats with DEN-Induced HCC

**Figure 3:**
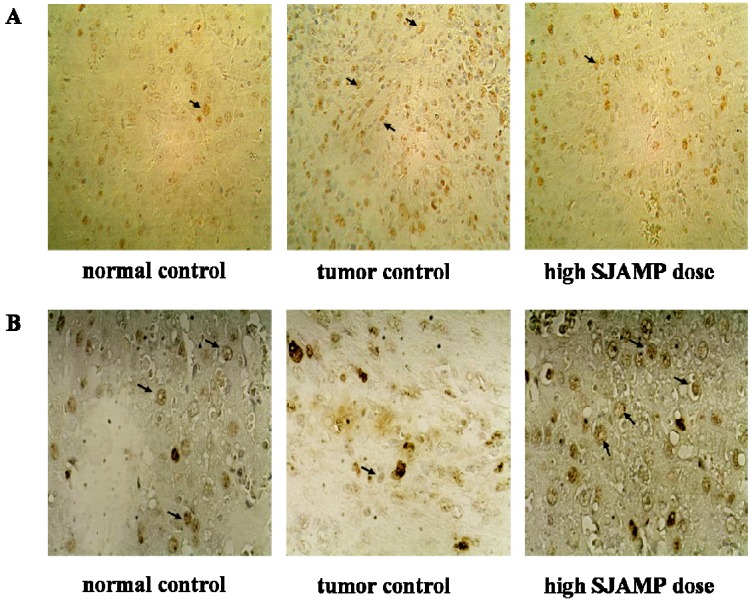
Representative photographs for immunohistochemical staining studies of PCNA and p21. (**A**) PCNA-stained cells (indicated with arrows) in normal control group, tumor control group and high-SJAMP-dose group (magnification factor × 200); (**B**) p21-stained cells (indicated with arrows) in normal control group, tumor control group and high-SJAMP-dose group (magnification factor × 400).

The expression of PCNA and p21 was examined by immunohistochemistry (IHC) in livers of all groups. Figure 3 shows representative IHC staining results for proliferating cell nuclear antigen (PCNA) and p21. As shown in Table 4, the PCNA labeling index was significantly increased, whereas p21-positive cells were dramatically decreased in the DEN-induced tumor control group rats compared to rats in the normal control group (*p* < 0.05). Compared with the DEN-induced tumor control group, the PCNA labeling indices of the SJAMP intervention groups were decreased (*p* < 0.05), while p21-positive cells of the SJAMP intervention groups were increased (*p* < 0.05) in a clear dose-response manner. 

**Table 4:** Effect of SJAMP on PCNA labeling index and p21-positive cells in rats with DEN-induced HCC (x ± SD).

Groups	Number (n)	PCNA (%)	P21 (%)
Normal control	10	30.30 ± 5.46	35.43 ± 4.20
Tumor control	7	59.71 ± 3.35 ^a^	16.40 ± 3.78 ^a^
Low SJAMP dose (17.5 mg/kg)	9	50.56 ± 3.64 ^b^	20.30 ± 2.50 ^b^
Medium SJAMP dose (35 mg/kg)	9	46.11 ± 3.72 ^b^	25.00 ± 2.35 ^b^
High SJAMP dose (70 mg/kg)	10	42.00 ± 3.65 ^b^	29.44 ± 2.88 ^b^

^a^ *p* < 0.05 *vs.* normal control group; ^b^ *p* < 0.05 *vs.* tumor control group.

Abnormal cell proliferation is the main feature of carcinogenesis, making inhibition of the excessive proliferation of tumor cells is an effective treatment approach. PCNA, a marker of cell proliferation, is associated with the DNA synthesis phase of the cell cycle [21,22]. p21, a tumor suppressor protein, exerts an inhibitory effect upon cell growth by binding to PCNA [23]. Our results demonstrated an anti-proliferative effect of SJAMP in hepatic tissue, which may, in part, account for its inhibitory effects on DEN-induced hepatocarcinogenesis.

### 2.5. The Effect of SJAMP on Immune Organs in Rats with DEN-Induced HCC

Mean immune organ indices are shown in Table 5. The mean thymus index of the tumor control group was significantly lower than in the normal control group (*p* < 0.05), but the mean thymus indices in the SJAMP intervention groups were higher than the tumor control group in a clear dose-response manner. Although the mean thymus index of the low-SJAMP-dose group was increased compared to the tumor control group, the increase did not reach statistical significance. The mean spleen index of the tumor control group was significantly higher than that of the normal control group (*p* < 0.05), indicating that the rats could mobilize their own immune organs to defend against external toxins when attacked by external challenges. The mean spleen indices in the SJAMP intervention groups were higher than in the tumor control group in a clear dose-response manner. There were no significant differences in these indices between the low-SJAMP-dose group and the tumor control group, however. Collectively, these results suggest that SJAMP can stimulate and promote growth of the spleen and thymus.

**Table 5:** Effect of SJAMP on thymus and spleen indices in rats with DEN-induced HCC (x ± SD).

Groups	Number (n)	Thymus index	Spleen index
Normal control	10	0.965 ± 0.159	1.165 ± 0.226
Tumor control	7	0.571 ± 0.167 ^a^	3.364 ± 0.770 ^a^
Low SJAMP dose (17.5 mg/kg)	9	0.706 ± 0.199 ^a^	3.491 ± 0.991 ^a^
Medium SJAMP dose (35 mg/kg)	9	0.928 ± 0.225 ^b^	4.418 ± 0.601 ^ab^
High SJAMP dose (70 mg/kg)	10	0.994 ± 0.173 ^b^	5.569 ± 0.969 ^ab^

^a^ *p* < 0.05 *vs.* normal control group; ^b^ *p* < 0.05 *vs.* tumor control group.

### 2.6. The Effect of SJAMP on Phagocytosis by Spleen Macrophages in Rats with DEN-Induced HCC

As shown in Table 6, the phagocytic activity of macrophages was improved in all of the tumor control and SJAMP intervention groups compared to the normal control group, and the SJAMP intervention groups showed the highest macrophage phagocytic activity (*p* < 0.01). Although the increase in the macrophage phagocytic activity in the low-SJAMP-dose group did not reach statistical significance compared to the tumor control group, there did appear to be a dose-response relationship between SJAMP intervention dose and phagocytic activity of the macrophages. Phagocytosis is one of the most important functions of macrophages in anti-tumor immunity [24]. Our results suggest that SJAMP could enhance immune function in rats with DEN-induced HCC. 

**Table 6:** Effect of SJAMP on the phagocytic activity of macrophage and the cytotoxic activity of NK cells in rats with DEN-induced HCC (x ± SD).

Groups	Number (n)	Phagocytic activity of macrophages (OD value)	Cytotoxic activity of NK cells (%)
Normal control	10	0.078 ± 0.003	40.8 ± 5.7
Tumor control	7	0.122 ± 0.012 ^a^	22.3 ± 10.4 ^a^
Low SJAMP dose (17.5 mg/kg)	9	0.125 ± 0.008 ^a^	25.1 ± 4.3 ^a^
Medium SJAMP dose (35 mg/kg)	9	0.139 ± 0.015 ^ab^	29.0 ± 4.5 ^ab^
High SJAMP dose (70 mg/kg)	10	0.144 ± 0.011 ^ab^	41.3 ± 6.9 ^b^

^a^ *p* < 0.01 *vs.* normal control group; ^b^ *p* < 0.01 *vs.* tumor control group.

### 2.7. The Effect of SJAMP on the Cytotoxic Activity of Natural Killer Cells in Rats with DEN-Induced HCC

As shown in Table 6, while the cytotoxic activity of NK cells in the DEN-induced HCC tumor control group was significantly lower than in the normal control group (*p* < 0.01), the cytotoxic activity of the NK cells in the low-SJAMP-dose group and high-SJAMP-dose group was significantly higher than that of the tumor control group (*p* < 0.01). Although the cytotoxic activity of the NK cells in the low-SJAMP-dose group was increased compared with the tumor control group, the increase did not reach statistical significance. Cytotoxic activity of the NK cells was therefore only restored by higher doses of SJAMP.

As the first line of immune defense against developing tumors, NK cells play an important role in immune surveillance for cancer cells, as they rapidly recognize and lyse a large variety of tumor cells without the help of either prior sensitization or MHC-dependent recognition [25,26,27]. Our data suggest that SJAMP was an important biological-response modifier of NK cells *in vivo*.

### 2.8. The Effect of SJAMP on Peripheral Blood T-Lymphocyte Subsets in Rats with DEN-Induced HCC

**Figure 4:**
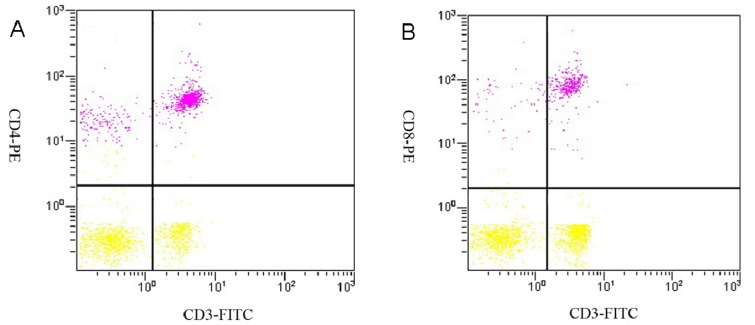
Examples of scatterplots presenting CD3^+^, CD4^+^ and CD8^+^ lymphocyte subsets. **(A**) Flow cytometric analysis of staining for CD3^+^ (x-axis) and CD4^+^ (y-axis) cells in the tumor control group. The CD3^+^ cells are represented in lower right quadrant, and the CD4^+^ cells are represented in the upper left quadrant. The CD3^+^CD4^+^ cells are represented in the upper right quadrant; (**B**) Flow cytometric analysis of staining for CD3^+^ (x-axis) and CD8^+^ (y-axis) cells in the tumor control group. The CD3^+^ cells are represented in the lower right quadrant, and the CD8^+^ cells are represented in the upper left quadrant. The CD3^+^CD8^+^ cells are represented in the upper right quadrant.

Peripheral blood T-lymphocyte subsets were analyzed using flow cytometry, and representative results from the tumor control group rats are shown in Figure 4. The percentage of T lymphocytes from each subset and the CD4^+^/CD8^+^ ratios are shown in Table 8. The CD3^+^ and CD4^+^ T lymphocyte levels and CD4^+^/CD8^+^ ratio in the tumor control group were significantly decreased compared to the normal control group (*p* < 0.05), but the level of CD8^+^ T lymphocytes was not significantly decreased (*p* > 0.05). With increasing dose of SJAMP, CD3^+^, CD4^+^ and CD8^+^ cell levels significantly increased, and the ratio of CD4^+^ to CD8^+^ cells also tended to normalize.

CD3^+^ is expressed on the surface of mature T lymphocytes. Mature T lymphocytes in the peripheral blood can be divided into CD4^+^CD8^−^ and CD4^−^CD8^+^ subgroups according to markers on the cell surface. CD4^+^ T cells are thought to play an important role in anti-tumor immunology by regulating the differentiation and development of CD8^+^ T cells. Immune regulation relies mainly on CD4^+^ T cells and CD8^+^ T cells, and dynamic changes in the CD4^+^/CD8^+^ ratio are regarded as an important factor in determining immunity states [13,28]. In the present study, we observed that CD4^+^ and CD8^+^ cells experienced significant recoveries and the ratio of CD4^+^ T cells to CD8^+^ T cells tended to normalize after SJAMP treatment, suggesting that SJAMP is capable of improving immune function in these rats.

**Table 8:** Effect of SJAMP on peripheral blood T-lymphocyte subsets in rats with DEN-induced HCC (x ± SD).

Groups	CD3^+^ (%)	CD4^+^ (%)	CD8^+^ (%)	CD4^+^/CD8^+^
Normal control	56.078 ± 10.202	35.200 ± 6.165	19.578 ± 5.398	1.909 ± 0.650
Tumor control	34.150 ± 12.761 ^a^	18.367 ± 11.702 ^a^	15.783 ± 5.010	1.286 ± 1.103 ^a^
Low SJAMP dose (17.5 mg/kg)	42.671 ± 10.676 ^a^	23.657 ± 9.664 ^a^	18.991 ± 9.969	1.663 ± 1.208
Medium SJAMP dose (35 mg/kg)	49.250 ± 8.925 ^b^	29.288 ± 7.810 ^ab^	19.912 ± 7.544	1.719 ± 0.905 ^b^
High SJAMP dose (70 mg/kg)	50.288 ± 11.013 ^b^	29.38 ± 12.851 ^ab^	20.810 ± 13.671 ^b^	1.890 ± 1.145 ^b^

^a^ *p* < 0.05 *vs.* normal control group; ^b^ *p* < 0.05 *vs.* tumor control group.

### 2.9. The Effect of SJAMP on Production of Serum Cytokines in Rats with DEN-Induced HCC

Serum levels of IL-2 and TNF-α in each group are shown in Table 9. Serum IL-2 level was significantly decreased, whereas TNF-α level was found to be dramatically increased in the DEN-induced tumor control group rats compared to rats in the normal control group (*p* < 0.01). The medium and high SJAMP doses significantly increased serum IL-2 and decreased serum TNF-α compared to the tumor control group (*p* < 0.01). The low SJAMP dose also increased serum IL-2 and decreased serum TNF-α compared with the tumor control group, but these changes did not reach statistical significance.

IL-2 is mainly secreted by CD4^+^ T cells. As a T cell growth factor and stimulator of NK cells, IL-2 augments the host’s cell-mediated anti-tumor immune response [29,30]. In the present study, we observed that serum IL-2 levels tended to normalize after SJAMP treatment, suggesting that SJAMP is capable of stimulating the T cell and NK cell-mediated immune responses in rats with DEN-induced HCC through enhancement of the production of IL-2. 

TNF-α is produced by many cell types including macrophages, CD4^+^ T cells, neutrophils, mast cells, fibroblasts, and tumor cells. As a potent pleiotropic proinflammatory cytokine, TNF-α affects the growth, differentiation, and survival of all cells [31]. Existing evidence indicates that high levels of TNF-α can favor cell survival and tumor progression [32]. In the present experiment, there was a marked increase in serum TNF-α level in DEN-induced tumor control group animals. This finding is consistent with a previous report that DEN-induced HCC in Wistar rats led to an increase in serum TNF-α [33]. The reduced level of TNF-α in rats with DEN-induced HCC by the administration of SJAMP may be related to its anti-tumor activity.

**Table 9:** Effect of SJAMP on serum cytokines production in rats with DEN-induced HCC (x ± SD).

Groups	Number (n)	IL-2 (pg/mL)	TNF-α (pg/mL)
Normal control	10	451.59 ± 38.15	11.857 ± 6.929
Tumor control	7	107.03 ± 21.21 ^a^	148.694 ± 22.186 ^a^
Low SJAMP dose (17.5 mg/kg)	9	119.00 ± 23.88	137.189 ± 17.133
Medium SJAMP dose (35 mg/kg)	9	301.93 ± 17.73 ^b^	99.310 ± 18.583 ^b^
High SJAMP dose (70 mg/kg)	10	370.50 ± 34.55 ^b^	92.136 ± 18.943 ^b^

^a^ *p* < 0.01 *vs.* normal control group; ^b^ *p* < 0.01 *vs.* tumor control group.

## 3. Experimental

### 3.1. Materials

A total of 50 Wistar mice (SPF grade, male, weighing 130–150 g) were purchased from the Shandong Lukang Laboratory Animal Center (Jining, China, SLXK-Lu20080002). SJAMP (purity > 99.5%) was provided by the College of Food Science and Engineering, Ocean University of China. The YAC-1 cell line was purchased from the Shanghai Cell Bank of the Chinese Academy of Sciences. Optilyse C lysing solution was purchased from Immunotech (Marseille, France). FITC anti-rat CD3 antibody, PE anti-rat CD4 antibody and PE anti-rat CD8a antibody were purchased from Biolegend (San Diego, CA, USA). TNF-α and IL-2 enzyme-linked immunoabsorbent assay (ELISA) kits were obtained from Boster Biological Technology (Wuhan, China). Mouse anti-PNCA was purchased from ZSGB-BIO (Beijing, China). Mouse anti-p21 was purchased from Thermo (Waltham, MA, USA).

### 3.2. Experimental Schedules

Fifty rats were randomly divided into a normal control group, tumor control group, low-SJAMP-dose group, medium-SJAMP-dose group, and high-SJAMP-dose group (10 rats/group). All animal experiments were performed according to the rules and regulations of the Animal Ethics Committee of Qingdao University Medical College, China. Rats in the normal control group were fed a normal diet. Rats in the tumor control group and SJAMP intervention groups received an intragastric administration of a 2% DEN saline solution (10 mg/kg of body weight) once daily for 5 days/week for a period of 15 weeks. Concurrently, the low-, medium- and high-SJAMP-dose groups were given 17.5, 35, and 70 mg/kg/day of SJAMP, respectively. The tumor control group received intragastric administration of an equivalent amount of normal saline. Rats were weighed once a week. At the 16^th^ week, all rats were anesthetized with chloral hydrate, and the abdominal aortic blood, liver tissues, spleen and thymus were separated and weighed.

Macroscopically visible nodules greater than 3 mm or 5 mm in diameter on the liver surface were recorded. The longest and shortest diameters of the largest tumor nodules were measured using a vernier caliper. Volume of the largest nodules was estimated using the following equation [34]:

nodule volume (mm^3^) = π/6 × a × b^2^ (1)

where a is the longest axis, and b is the shortest axis).

### 3.3. Measurement of Liver Function Parameters, Serum AFP, TNF-α and IL-2 Levels

Levels of ALT, AST and GGT in each serum sample were measured using a Hitachi 7170A Modular Analytic system. Serum concentrations of AFP, TNF-α and IL-2 were assayed by the ELISA method using commercially available enzyme immunoassay kits, according to the manufacturer specifications.

### 3.4. Pathological Histology and Immunohistochemistry

Liver tissues were collected, embedded in paraffin, cut into slices and stained with hematoxylin and eosin (H & E) for histopathological examination under a microscope. Paraffin-embedded, 5 μm-thin sections from liver specimens were deparaffinized, blocked with 0.3% hydrogen peroxide for 10 min and subjected to antigen retrieval in a steamer for 20 min. The slides were then washed twice with PBS and incubated with primary antibodies of PCNA and p21. Incubation with an appropriate secondary antibody was followed by direct DAB staining and light counterstaining with hematoxylin. Staining procedures strictly followed the supplier’s recommendations. The proliferation index was determined as the percentage of positively stained nuclei by counting 1000 cells in field at magnification × 200. p21 was quantified by counting positively stained cells and total number of cells at 10 randomly selected fields from each section at 400 × magnifications, and data are presented as a percentage.

### 3.5. Preparation of Spleen Cell Suspensions

The spleens of the experimental animals were washed twice with D-Hank’s solution to remove blood and other non-specific tissues. Approximately 0.5 cm^3^ of spleen was split and ground, and the spleen cells were resuspended. Cells were washed twice with D-Hank’s solution, and the supernatants were discarded. Five mL of an NH_4_Cl lysis solution was then added to lyse the erythrocytes. After centrifuging the suspensions at 1,000 rpm for 15 min at 4 °C, the remaining cells were washed twice with PBS and then resuspended in RPMI 1640 medium supplemented with 10% fetal calf serum. The cells were then seeded into culture bottles and cultured for 2 h in a CO_2_ incubator at 5% CO_2_. The vast majority of the adherent cells were macrophages, and the non-adherent cells were mainly mixed spleen lymphocytes.

### 3.6. Determination of the Phagocytic Activity of the Macrophages

Phagocytic ability of macrophages was measured via neutral red dye uptake [35]. Adherent macrophages were released into single-cell suspensions using a 0.25% trypsin digestion and adjusting the cell density to 1 × 10^6^ cells/mL. An aliquot of 200 μL of the cell suspension was added to each of the wells of a 96-well plate (each sample had three parallel wells) and cultured for 48 h. One hundred μL of the culture medium was then aspirated from each well, 100 μL of a 0.1% neutral red solution was added to each well, and the plate was incubated for 30 min at 37 °C in a 5% CO_2_ humidified incubator. Finally, the plate was washed three times with PBS, and 100 μL of lysis buffer (0.1 mol/L acetic acid:ethanol = 1:1) was added to each well. The treated plates were stored at 4 °C in the refrigerator overnight, and the OD value at 492 nm was determined using an enzyme-labeled instrument.

### 3.7. Assessment of NK Cell Cytotoxicity

A lactate-dehydrogenase (LDH)-release assay was used to measure the NK cell-mediated tumor cytotoxicity. Spleen cells from each rat, prepared as previously described, were used as the effector cells. The NK-sensitive cell line YAC-1 was used as the target cell. Two types of cell suspension were prepared that contained 95% viable cells based on staining determinations with trypan blue. A 200 μL cell suspension that including 100 μL of the effector cell suspension (1 × 10^6^/mL) and 100 μL of the target cell suspension (1 × 10^5^/mL) was added into the wells of 96-well plates (each sample had three parallel wells). After a 4-h incubation at 37 °C in a 5% CO_2_ humidified incubator, the plates were centrifuged at 1,500 rpm for 15 min. The supernatants from each well (100 μL) were transferred into the corresponding wells of another 96-well plate. One hundred μL of lactic acid hydrogenase substrate mixture was then added to each well. After 6 min, the reactions were stopped with 1 mol/L HCl. The OD value of each well was measured at 492 nm. The cytotoxic activity of the NK cells was estimated using the following equation:

cytotoxicity (%) = [(*E−S*)/(*M−S*)] × 100% (2)

where *E* represents the experimental release of LDH activity from the target cells incubated in the presence of the effector cells, *S* is the spontaneous release of the LDH activity from the target cells alone, and *M* represents the maximum release of the LDH activity determined by lysing the target cells with 1% NP-40.

### 3.8. T-lymphocyte Subsets in Rat Peripheral Blood Flow Cytometric Analyses

Aliquots of 100 μL of peripheral blood from each rat were added to Eppendorf tubes containing pre-added monoclonal antibodies against CD3, CD4 and CD8a (anti-CD3-FITC, anti-CD4-PE and anti-CD8-PE) and incubated for 15 min at room temperature in the dark. The red blood cells were lysed using Optilyse C lysing solution following the manufacturer’s instructions. After centrifuging at 1,000 rpm for 10 min at room temperature in the dark, the samples were washed twice with PBS. Then, the cell sediments were suspended in 0.5 mL PBS. The prepared suspensions were analyzed on a Cytomics FC 500 flow cytometer (Beckman Coulter). A total of 20,000 cells were analyzed for each sample.

### 3.9. Statistics

All data are expressed as the means ± SD. The data were statistically analyzed using one-way analysis of variance (ANOVA). *Post-hoc* comparisons were carried out by the least-significant difference (LSD) test. Probability values lower than 0.05 were considered significant.

## 4. Conclusions

In summary, our results show that SJAMP was effective against HCC. The anti-tumor mechanisms of SJAMP may be characterized as protecting the immune organs, stimulating the proliferation of the tissues from the immune organs and enhancing cell-mediated immunity. 
